# Editorial: Human and Oncoviral Non-Coding RNAs as Modulators of Cancer Aggressiveness and Disease Progression

**DOI:** 10.3389/fonc.2020.641725

**Published:** 2021-01-25

**Authors:** Patricia P. Reis, Wan L. Lam, Deilson Elgui de Oliveira

**Affiliations:** ^1^ Medical School, São Paulo State University (UNESP), Botucatu, Brazil; ^2^ University of British Columbia, Vancouver, BC, Canada

**Keywords:** non-coding RNAs, cancer biology, tumor progression, oncoviruses, cancer management, human

Non-coding RNAs (ncRNAs) include a variety of molecules that modulate complex cellular networks with roles in normal physiology and cancer pathogenesis (1). Several types of ncRNAs, such as microRNAs (miRNAs) and long non-coding RNAs (lncRNAs)—either endogenous or from viral origin—have been widely investigated in human cancers. They are diagnostic, prognostic, and predictive biomarkers, detectable in biofluids (2). Human and viral ncRNAs contribute to the hallmarks of cancer, modulating target genes participating in disease development and tumor progression (3–6).

This Research Topic compiles original research and review papers that shed light on the role of human or viral ncRNAs in the molecular and cellular processes leading to cancer progression. The ncRNAs orchestrate malignant invasion and the dissemination of neoplastic cells, notably by metastasis, by targeting genes within several signaling pathways, such as those in the Epithelial-Mesenchymal Transition program. The 13 papers on this Research Topic highlight ncRNAs as key players in cancer pathogenesis, some proposed as biomarkers applicable to clinical management of patients.

Mou et al. showed that the lncRNA *Lymph Node Metastasis Associated Transcript 1* (*LNMAT1*) is upregulated in malignant melanoma. Mechanistically, *LNMAT1* enhances cancer cell migration and invasion by suppressing the *Cell Adhesion Molecule 1* (*CADM1*) tumor suppressor gene. In colorectal carcinoma (CRC), Lin et al. demonstrated that the *miR-506* targets the epigenetic modifier UHRF1 *via* the KISS1/PI3K/NF-κB signaling axis, inhibiting the proliferation, migration, and invasion of CRC cells *in vitro* and *in vivo*, suggesting new therapeutic targets for CRC. In gastric cancer (GC), Sun et al. showed that the lncRNA *AK025387* was upregulated in metastatic GC cells, putatively by regulation of the Raf-1/MAPK/MEK/ERK signaling. Zhao et al. reviewed the role of lncRNAs and miRNAs in regulating the *Programmed Cell Death 4* (*PDCD4*) tumor suppressor gene, which is involved in a variety of cellular regulatory mechanisms, to disclose relevant information for the development of novel therapies for targeting cancers disrupted in PDCD4 suppression.

Drugs and natural compounds have been widely explored regarding their activity and cytotoxicity against various cancers for decades. In this regard, Liu et al. reported that the natural glycoalkaloid Solasonine (SS) inhibited the growth of HepG2 and QGY-7703 hepatocellular carcinoma (HCC) cells, and this effect was attributed to regulatory interaction between miR-375-3p and the lncRNA CCAT1, leading to reduction of IRF5 mediated by the SP1 transcription factor. Yan et al. showed a novel mechanism of Temozolomide resistance in glioblastoma cells based on activity of lnRNA *ADAMTS9* and the AS2/FUS/MDM2 signaling axis. LncRNA alterations may lead to the acquisition of resistance to chemotherapy. Abildgaard et al. present the landscape of lncRNA alterations associated with the hallmarks of ovarian carcer (OC), concluding that OC pathogenesis and acquisition of treatment resistance may occur through highly complex mechanisms in which ncRNAs have prominent roles.

In chronic myeloid leukemia (CML), allogeneic-hematopoietic stem cell transplantation (allo-HSCT) may be the treatment of choice for patients with resistant forms of the disease. Martins et al. evaluated the miRNA expression profiles in CML in 28 patients equally divided into groups with untreated cases of chronic phase-CML and cases with cytogenetic remission after allo-HSCT. Among the differentially expressed miRNAs, the miR-1260a and miR-409-3p were identified as the top downregulated and upregulated miRNAs, respectively. Interestingly, the signaling pathways involving MAPKs, RAS, and ROCK were enriched with components encoded by genes targeted by these miRNAs, suggesting that they may have regulatory functions in the clinical evolution of CML.

A significant fraction of human cancers is linked to infection by oncogenic viruses, such as the Human Papilloma Virus (HPV) and the Epstein-Barr virus (EBV). Tornesello et al. describe complex interactions between miRNAs, lncRNAs, and circular RNAs (circRNAs) in the pathogenesis of cervical carcer strongly linked to HPV infection. The lncRNAs *HOTAIR*, *MALAT1*, *GAS5*, and *MEG3* were reported to regulate tumor invasion and therapeutic resistance in cervical cancer. Additionally, the circRNA, circ-0018289 contributes *via* a miRNA-sponging mechanism. This network of interacting ncRNAs may also include HPV microRNAs (e.g., HPV-16-miR-H1-1 and HPV-16-miR-H2-1), but further investigation of this matter is required to clarify their role in HPV-associated cancers. Nonetheless, viral ncRNAs encoded by EBV are already recognized as key players of EBV-induced carcinogenesis. Verhoeven et al. reported that the BART region of the EBV genome encodes lncRNAs that not only contribute to EBV latency in nasopharyngeal carcinomas (NPC) cells, but also regulate genes that ultimately may cause immune evasion and contribute to NPC progression.

The discovery of previously unannotated miRNAs in several tissue types (7) has prompted the identification of new ncRNAs with biological and clinical relevance in cancers. Rock et al. reported that 146 novel miRNAs specific to head and neck squamous cell carcinomas (HNSCC) were identified from small RNA sequence data *in silico*. Interestingly, 135/146 miRNAs were found increased in HNSCC samples compared to non-neoplastic head and neck tissues. The novel miRNAs identified were validated with HNSCC samples; furthermore, a prognostic-model combining novel and known miRNAs and using multivariate Cox regression analysis was proposed, allowing improved stratification of survival and recurrence risk of patients. In pancreatic cancer, Ros et al. reported new antisense lncRNAs associated with *High Mobility Group A* (*HMGA1* and *HMGA2*) genes, and *HMGA2-AS1* lncRNA regulates the expression of its own sense gene, with implications for tumorigenesis. Finally, Wu and Chen reviewed the role of super-enhancers (SE) molecules and se-ncRNAs, which may have potent activities as effectors in the determination of cell identity and their crosstalk with immune checkpoints. SEs associated with ncRNAs have shown to play a role in several regulatory mechanisms, including immune checkpoint expression in cancer cells, and the authors highlight a number of studies that explored the use of SE blockers for improved cancer therapies.

Altogether, these papers included in this Research Topic by Frontiers shed more light on the characterization of known and novel ncRNAs with relevant roles in cancer biology and that may be valuable as biomarkers to assess prognosis, response to treatment, and monitoring of patients with aggressive cancers.

## Author Contributions

All authors contributed equally to this Editorial. All authors contributed to the article and approved the submitted version.

## Conflict of Interest

The authors declare that the research was conducted in the absence of any commercial or financial relationships that could be construed as a potential conflict of interest.
